# SARS-COV-2 colonizes coronary thrombus and impairs heart microcirculation bed in asymptomatic SARS-CoV-2 positive subjects with acute myocardial infarction

**DOI:** 10.1186/s13054-021-03643-0

**Published:** 2021-06-24

**Authors:** Raffaele Marfella, Pasquale Paolisso, Celestino Sardu, Luciana Palomba, Nunzia D’Onofrio, Arturo Cesaro, Michelangela Barbieri, Maria Rosaria Rizzo, Ferdinando Carlo Sasso, Lucia Scisciola, Fabrizio Turriziani, Massimiliano Galdiero, Danilo Pignataro, Fabio Minicucci, Maria Consiglia Trotta, Michele D’Amico, Ciro Mauro, Paolo Calabrò, Maria Luisa Balestrieri, Giuseppe Signioriello, Emanuele Barbato, Marilena Galdiero, Giuseppe Paolisso

**Affiliations:** 1 Department of Advanced Medical and Surgical Sciences, University of Campania “Luigi Vanvitelli” Naples, Piazza Miraglia, 2, 80138 Naples, Italy; 2 Mediterranea Cardiocentro, Naples, Italy; 3 Department of Advanced Biomedical Sciences, University of Naples Federico II, Naples, Italy; 4 Department of Experimental Medicine, University of Campania “Luigi Vanvitelli”, Naples, Italy; 5 Department of Precision Medicine, University of Campania “Luigi Vanvitelli” Naples, Naples, Italy; 6 Department of Cardio-Thoracic and Respiratory Sciences, University of Campania “Luigi Vanvitelli”, Naples, Italy; 7 Department of Cardiology, Hospital Cardarelli, Naples, Italy; 8 Department of Experimental Medicine, University of Campania “Luigi Vanvitelli”, Naples, Italy; 9 Cardiovascular Center Aalst, Aalst, Belgium; 10 Department of Mental Health and Preventive Medicine, University of Campania “Luigi Vanvitelli”, Naples, Italy

**Keywords:** Intracoronary thrombus, SARS-COV-2, Thrombus viral load, Asymptomatic SARS-COV-2 patients, STEMI

## Abstract

**Background:**

The viral load of asymptomatic SAR-COV-2 positive (ASAP) persons has been equal to that of symptomatic patients. On the other hand, there are no reports of ST-elevation myocardial infarction (STEMI) outcomes in ASAP patients. Therefore, we evaluated thrombus burden and thrombus viral load and their impact on microvascular bed perfusion in the infarct area (myocardial blush grade, MBG) in ASAP compared to SARS-COV-2 negative (SANE) STEMI patients.

**Methods:**

This was an observational study of 46 ASAP, and 130 SANE patients admitted with confirmed STEMI treated with primary percutaneous coronary intervention and thrombus aspiration. The primary endpoints were thrombus dimension + thrombus viral load effects on MBG after PPCI. The secondary endpoints during hospitalization were major adverse cardiovascular events (MACEs). MACEs are defined as a composite of cardiovascular death, nonfatal acute AMI, and heart failure during hospitalization.

**Results:**

In the study population, ASAP vs. SANE showed a significant greater use of GP IIb/IIIa inhibitors and of heparin (*p* < 0.05), and a higher thrombus grade 5 and thrombus dimensions (*p* < 0.05). Interestingly, ASAP vs. SANE patients had lower MBG and left ventricular function (*p* < 0.001), and 39 (84.9%) of ASAP patients had thrombus specimens positive for SARS-COV-2. After PPCI, a MBG 2–3 was present in only 26.1% of ASAP vs. 97.7% of SANE STEMI patients (*p* < 0.001). Notably, death and nonfatal AMI were higher in ASAP vs. SANE patients (*p* < 0.05). Finally, in ASAP STEMI patients the thrombus viral load was a significant determinant of thrombus dimension independently of risk factors (*p* < 0.005). Thus, multiple logistic regression analyses evidenced that thrombus SARS-CoV-2 infection and dimension were significant predictors of poorer MBG in STEMI patients. Intriguingly, in ASAP patients the female vs. male had higher thrombus viral load (15.53 ± 4.5 vs. 30.25 ± 5.51 CT; *p* < 0.001), and thrombus dimension (4.62 ± 0.44 vs 4.00 ± 1.28 mm^2^; *p* < 0.001). ASAP vs. SANE patients had a significantly lower in-hospital survival for MACE following PPCI (*p* < 0.001).

**Conclusions:**

In ASAP patients presenting with STEMI, there is strong evidence towards higher thrombus viral load, dimension, and poorer MBG. These data support the need to reconsider ASAP status as a risk factor that may worsen STEMI outcomes.

## Introduction

Since February 2020, there have been reports of infected persons with SARS-CoV-2 but did not develop symptoms of coronavirus disease 2019 (COVID-19) [1–3]. Asymptomatic people appear to account for about 40–45% of SARS-CoV-2 infections and may transmit the virus to others over a prolonged period, perhaps more than 14 days [1]. Moreover, the asymptomatic infection may be associated with subclinical lung abnormalities, as detected by computed tomography [4]. Interestingly, the viral load of such asymptomatic persons has been frequently found equal to that of symptomatic persons [5, 6], thus suggesting a similar risk for endothelial dysfunction and increased coagulation in asymptomatic and symptomatic patients. The role of SARS-COV-2 in leading endothelial dysfunction and increased coagulation is also well known in patients with acute myocardial infarction (AMI) [7]. In fact, patients presenting with ST-Elevation Myocardial Infarction (STEMI) and concurrent COVID-19 infection evidenced a strong signal towards higher thrombus burden and poorer outcomes [7].

Moreover, these data are supported by the observations that the first AMI incidence was ≈5 times higher during the acute phase of COVID-19 infection than the control [8]. However, this study did not provide any evidence about the potential role of SARS-COV-2 infection on thrombus viral load and thrombus burden neither on clinical outcomes in asymptomatic SAR-COV-2 positive (ASAP) STEMI patients compared to SARS-COV-2 negative (SANE) STEMI patients. In this context, we hypothesized that SAR-COV-2 thrombus viral load, by increasing coagulative state and coronary thrombus dimension of culprit atherosclerotic lesion, may worsen clinical outcomes in ASAP patients presenting STEMI. Therefore, we evaluated thrombus viral load and thrombus burden and their impact on microvascular bed perfusion in the infarct area (Blush grade) in consecutive ASAP cases compared to SANE STEMI patients.

## Methods

### Patient characteristics, angiographic and echocardiographic procedures evaluations

This was a multicenter observational cohort study aimed to investigate the relationship between thrombus viral load, thrombus dimension, and in-hospital outcomes in ASAP STEMI patients. We examined patients with first STEMI treated with the primary percutaneous coronary intervention (PPCI) and thrombus aspiration (TA) without coronavirus disease 2019 (COVID-19) between February 2020 and November 2020. Patients with clinical evidence of COVID-19 were excluded from the study. All patients met the guideline definition of STEMI [9]. Routine analyses were obtained on admission before coronary angiography and before the initiation of full medical therapy. All patients with STEMI had baseline serological samples before cardiac catheterization for full blood count, renal and liver function tests, C-reactive protein, D-dimer, fibrinogen, lactate dehydrogenase (LDH), and high sensitivity (hs)-Troponin T. We considered eligible for the study all patients with: correspondence between ECG findings and suspected culprit artery; a minimum visual estimate of 50% stenosis in the culprit artery, and feasibility of performing TA, as judged by the treating physician; the age of 18 years or greater; presentation to the cardiac catheterization laboratory for PPCI in the setting of first STEMI. Patients with left ventricular ejection fraction less than 25%, with previous myocardial infarction or previous PPCI and/or coronary by-pass grafting, or who had received fibrinolytic therapy were instead excluded from the study.

#### Coronary angiography

Coronary angiography was performed either via the radial or femoral artery. The culprit lesion was identified and crossed with an angioplasty guidewire. During primary PCI, unfractionated heparin was administered in a loading dose of 70–100 U/kg with the activated clotting time (ACT) maintained > 250 s. ACTs were recorded at 10–15 min intervals after the initial dose of heparin. Glycoprotein (GP) IIb/IIIa inhibitors were used at the operator’s discretion.

#### Thrombus burden

The thrombus content was classified by a modified TIMI Thrombus Grade Classification [10]. This classification scores the thrombus in five grades: Grade 0 (G0): No angiographic characteristics of thrombus are present; Grade 1 (G1): Possible thrombus is present (reduced contrast density, haziness, or irregular lesion contour); Grade 2 (G2): There is definite thrombus, with greatest dimensions ≤ half the vessel diameter; Grade 3 (G3): Definite thrombus, with greatest linear dimension > half the vessel diameter but < 2 vessel diameters; Grade 4 (G4): Definite thrombus, with the largest dimension ≥ 2 but < 4 vessel diameters; Grade 5 (G5): Definite thrombus, with the largest dimension ≥ 4 vessel diameters [2]. To date, two experienced interventional cardiologists independently evaluated all angiographic parameters. Two independent pathologists, blinded to study protocol, evaluated the thrombus dimension. After TA, thrombus surface area was defined as the product of its length, height, and thickness. Therefore, the thrombus dimension was expressed as surface area in mm^2^.

#### Thrombus aspiration

2017 Guidelines of the European Society of Cardiology for the management of acute myocardial infarction in patients presenting with ST-segment elevation [11] and 2018 Guidelines on myocardial revascularization [12] do not recommend routine use of thrombus aspiration (class III, level A). Nevertheless, the same guidelines state that thrombus aspiration may be considered in large residual thrombus burden cases. According to these recommendations, with the support of the flowchart proposed by Junhua Ge et al. [13], manual TA was performed based on angiographic selection criteria (e.g., the presence of a visible thrombus on angiography, large vessel easy to pass with the catheter, localization of the thrombus at the proximal or middle segments of the target vessel, TIMI Thrombus Grade Classification Grade G3–G5), followed by conventional PCI to the culprit's vessel. The TA started before crossing the lesion, with a minimum of two syringes (40 mL) of aspirate recommended. Investigators were appropriately trained to ensure that the guide catheter was engaged with the coronary ostia when removing the thrombectomy catheter. Finally, the guide catheter was aspirated after thrombectomy to avoid embolization of either air or thrombus from the guide catheter.

#### Myocardial blush grade

The Myocardial Blush Grade (MBG) was defined according to the Zwolle criteria [14]. Angiographic assessment of myocardial reperfusion in patients treated with primary angioplasty for acute myocardial infarction: myocardial blush grade. However, we defined the blush grade into grades from 0 to 3. Thus, grade 0 indicated no myocardial blush or contrast density; or persistent “staining,” suggesting leakage of the contrast medium into the extravascular space. The grade 1 blush was defined as minimal myocardial blush or contrast density. Grade 2 was indicative of moderate blush or obtained on the contralateral/ipsilateral non-IRA, and grade 3 was indicative of normal blush. The angiography studies were evaluated at 2 independent centers by experienced interventional cardiologists in both cases. The observers were blinded to the remaining clinical information. Laboratory 1 (Lab 1) is the institution that carried out the study, the catheterization laboratory of a teaching hospital, and laboratory 2 (Lab 2), an independent catheterization core laboratory with extensive experience measuring the myocardial blush index. Grades 2 and 3 were considered normal perfusion. The interobserver variability was 10% for detecting grades 2 and 3 blush and 20% for grade 3 blush in Lab 1, 13% and 15%, respectively, in Lab 2.

#### Echocardiographic evaluation

In the current study, two experienced physicians in echocardiography performed a trans-thoracic two-dimensional echocardiogram with M-mode, conventional Doppler imaging (TDI) measurements in each patient admitted for acute ST-elevation myocardial infarction (STEMI). For the examinations, we used a Philips iE33 echocardiography (Eindhoven, The Netherlands). The echocardiography was performed at hospital admission and at hospital discharge. Then, physicians acquired the images of echocardiography in the parasternal long and short-axis views. Thus, we calculated left ventricle end-diastolic diameter (LVEDD), end-diastolic volume (LVEDV), end-systolic diameter (LVESD), end-systolic volume (LVESV), and we determined left ventricle ejection fraction (LVEF) with the Simpson biplane method (ref). However, the physicians systematically performed averaged measurements in five consecutive samples to have final calculation measures. The physicians involved in the echocardiographic evaluation performed and analyzed each examination independently and blinded to the study protocol. Finally, two observers blinded to measures performed previously reviewed all measurements. The observers were blinded to study protocol [15].

The study follows the principles outlined in 1976, the Declaration of Helsinki, and its later amendments for the use of human tissue or subjects. The Institutional Review Board of University of Campania “Luigi Vanvitelli,” Naples, Italy approved the protocol (Ethical Committee number 268 for study on SARS-COV-2, and number 151 for study TA).

### Nasal/pharyngeal swab and thrombus SARS-COV-2 analysis

Post-catheterization, all patients underwent routine nasal/pharyngeal swab and thrombus analysis for the SARS-CoV-2 virus using real time-polymerase chain reaction (RT-PCR). The sample was dissolved by homogenization and then extracted by ripospinvrd kit (GeneAll). The RT-PCR was performed on CFX-96 Real-time PCR system (Bio-Rad) by Allplex 2019-nCoV assay, based on the analysis of four fluorophores: FAM for the revelation of E gene, Cal Red 610 for RdRP gene, Quasar 670 for N gene, and HEX to analyze the internal control (IC). The result was evaluated through Seegene Viewer. Respiratory and thrombus specimens were collected by the local CDC and then shipped to designated authoritative laboratories to detect SARS-CoV-2 presence and load.

The viral load has been detected with cycle threshold (CT) values [5, 16]. CT values are the number of cycles needed to detect each genetic marker identified by real-time reverse transcription-polymerase chain reaction testing. A lower CT value indicates a higher amount of viral RNA. Paired values for each resident are depicted using a different shape. Positivity was defined as a cycle threshold (CT) value ≤ 38.0. Based on the admission SARS-CoV-2 RNA nasopharyngeal swab, patients were clustered in two groups: ASAP STEMI patients (positive nasopharyngeal swab) and SANE STEMI patients (negative nasopharyngeal swab). Patients with laboratory-confirmed SARS-CoV-2 infection were defined as asymptomatic, who has no symptoms at the time of first clinical assessment and had no symptoms at the end of follow-up. The end of follow-up was defined as one or more negative respiratory specimen RT-PCR test results.

### In-hospital outcomes

The primary endpoints were thrombus dimension + thrombus viral load effects on microvascular bed perfusion in the infarct area (Blush grade) after PPCI. The secondary endpoints during hospitalization were major adverse cardiovascular events (MACEs) defined as a composite of cardiovascular death, nonfatal acute AMI, and heart failure during hospitalization.

### Statistical analysis

Descriptive statistical analyses were performed using SPSS Statistics version 23.0 (IBM). A 2-sided *p* value < 0.05 defined statistical significance. Variables are expressed accounts (percentages), mean ± standard deviation (SD), and median [lower quartile-upper quartile] as appropriate. Chi-squared analysis or Fisher’s-exact test was used to compare categorical data between groups. The independent samples Student *t* test or ANOVA test was used to compare normally distributed continuous data between groups, and the Mann-Whitney *U* test was used to compare the distribution of continuous skewed data between groups.

Correlation performed using Pearson’s correlation analysis and Spearman’s correlation analysis in the case of skewed variables. Event rates were derived as Kaplan–Meier estimates and compared by log-rank test.

Transforming the MBG as as a dichotomic variable, indicating with 0 the MBG < 2 (MBG 0, and 1), and indicating as 1 the cut off of blush grade in the case of MBG > 2 (MBG 2, and 3) we build a multiple logistic regression analysis to evaluate the independent association of SARS-CoV-2 infection and thrombus viral load with thrombus dimension independently of age, sex, metabolic risk factors, hypertension, and smoking. Thus, in a first model (A) we tested this analysis for all study population (*n* = 176). Then, we build a second model (B) and third model (C) including in the analysis also the SARS-CoV-2 infection for B, and excluding the thrombus dimension from the analysis in the model C. The models B and C were built to evaluate the multiple logistic regression for ASAP patients (*n* = 46). The odds ratio and 95% confidence intervals (CI) were also calculated.

To date, the composite score for MACE has been build summarizing the variables as cardiovascular death, nonfatal acute AMI, and heart failure during hospitalization. Therefore, the MACE as study endpoint was diagnosed by the evidence at least of one of the indicated conditions (cardiovascular death, nonfatal acute AMI, and heart failure during hospitalization).

The sample size, estimated according to a global effect size of 27% with type I error of 0.05 and a power of 90%, was 140 patients. To date, we calculated a post hoc sample size for the study primary endpoint as global effect that was about 30 participants as ASAP and 110 participants as SANE.

## Results

### Patient characteristics

**Fig. 1:**
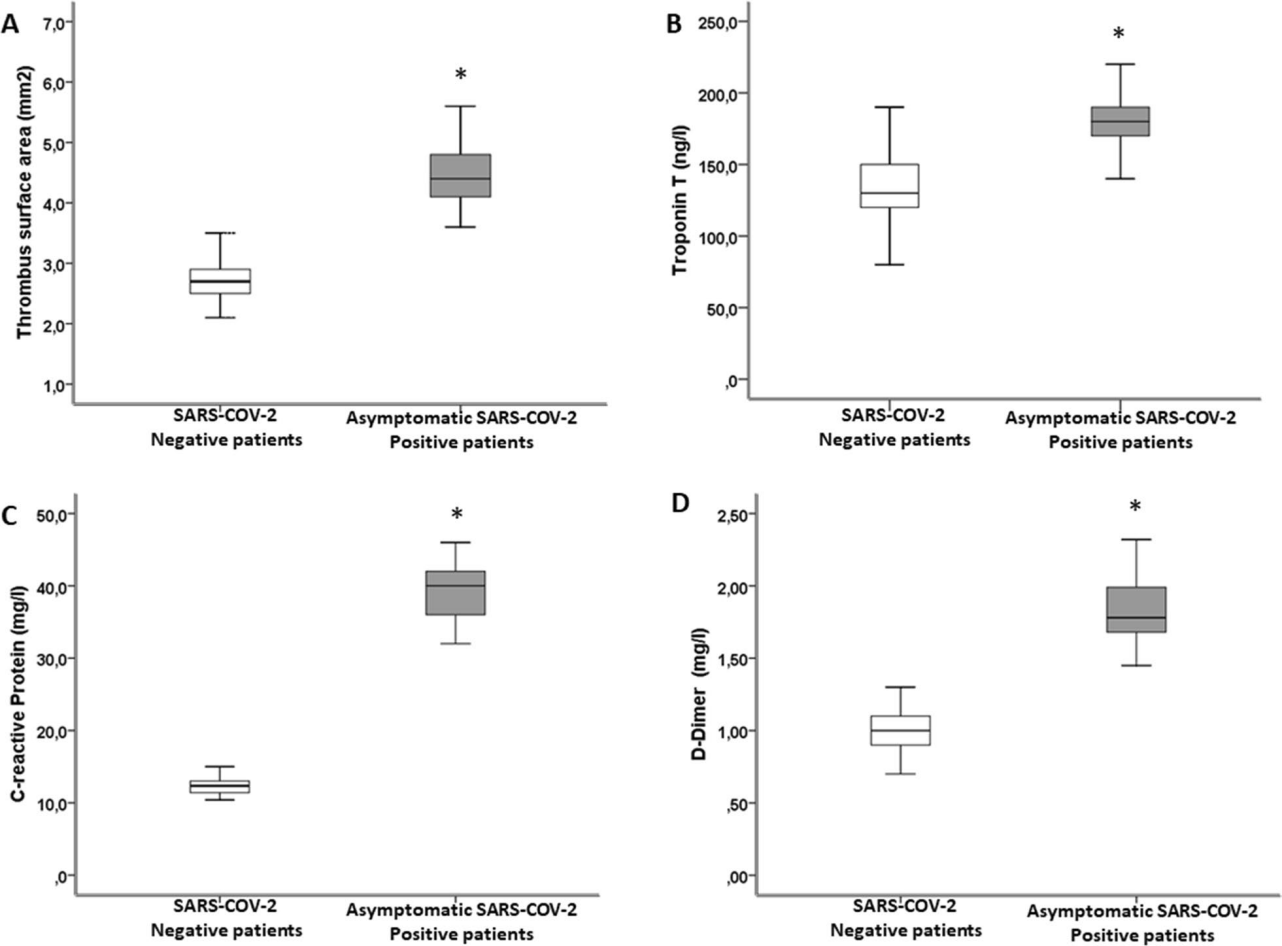
**a** Thrombus surface areas (mm^2^) from 130 SARS-COV-2 negative STEMI patients and 46 asymptomatic SARS-COV-2 positive STEMI patients (Boxplot, a plot type that displays the median, 25th, and 75th percentiles and range). **b** troponin T levels from 130 SARS-COV-2 negative STEMI patients and 46 asymptomatic SARS-COV-2 positive STEMI patients. **c** C-reactive protein levels from 130 SARS-COV-2 negative STEMI patients and 46 asymptomatic SARS-COV-2 positive STEMI patients. **d** D-dimer levels from 130 SARS-COV-2 negative STEMI patients and 46 asymptomatic SARS-COV-2 positive STEMI patients. Data are mean ± SD. *P < 0.01 vs. asymptomatic SARS-COV-2 positive STEMI patients.

**Fig. 2:**
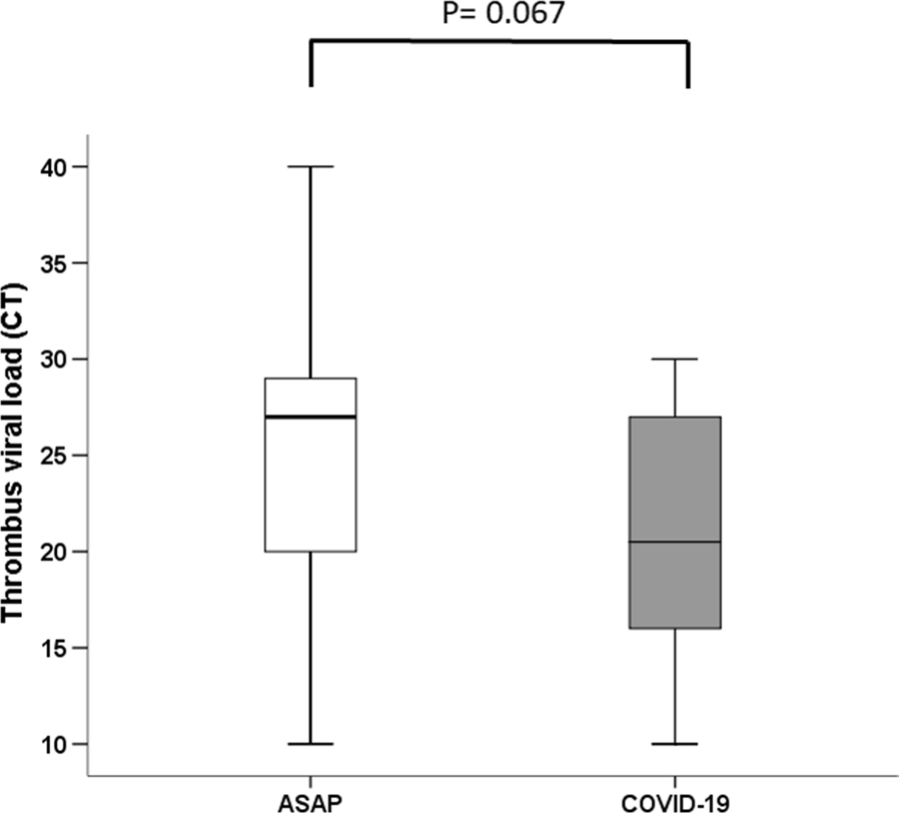
Cycle thresholds in thrombi from asymptomatic SARS patients and COVID-19 patients.

A total of 777 patients with acute myocardial infarction were admitted at cardiologic study centers between February 2020 and November 2020. Of these, 474 (61.1%) were hospitalized for STEMI and 303 for No ST-elevation myocardial infarction (NSTEMI). The normalization of myocardial injury markers was not different in the two groups. Consequently, 474 were considered eligible STEMI patients and underwent the angiographic study. After the coronary study, 8 patients were excluded due to CABG indication, 3 for the absence of coronary lesions, while 287 were not treated with TA. Thus, the study population consisted of 176 consecutive patients with confirmed STEMI, submitted to PPCI and TA, admitted during 40 weeks. Of the 176 patients, 46 (26.1%) were ASAP patients, while 130 (73.9%) patients were SANE patients. In ASAP patients, the viral load was 26 ± 8 CT. Interestingly, 39 (84.9%) of asymptomatic patients with SARS-COV-2 positive respiratory specimens had also thrombus specimens positive for SARS-COV-2. No statistical significant differences comparing SANE STEMI vs. ASAP STEMI regards diabetes (38 (29.2%) vs. 8 (17.4%); *p* = 0.478) were found. SANE STEMI vs. ASAP STEMI patients had higher rate of hypertension (72 (55.4%) vs. 18 (39.1%); *p* = 0.042), and they had higher body mass index (BMI) and age (Table 1). ASAP patients had higher thrombus dimension (A), hs-Troponin (B), D-dimer (C), and C-reactive protein (D) and levels than SANE patients (Fig. 1). Interestingly, we evaluated thrombus viral load in 12 patients asymptomatic at screening, but that developed COVID-19 during hospitalization. These patients were not included in the evaluation. The thrombus viral load was similar to the ASAP patients in these patients (Fig. 2).

**Table 1:** Clinical and instrumental characteristic of study population

	Asymptomatic SARS-COV-2 Positive patients (*N*= 46)	SARS-COV-2 Negative patients (*N* = 130)	*P*
Age, years	56.13 ± 6.21	68.43 ± 6.46	0.006
Male, *n* (%)	31 (67.4)	86 (66.2)	0.515
BMI (kg/m^2^)	27.09 ± 1.81	29.55 ± 1.97	0.003
Diabetes, *n* (%)	8 (17.4)	38 (29.2)	0.478
Dyslipidemia, *n* (%)	7 (15.2)	30 (23.7)	0.181
Hypertension, *n* (%)	18 (39.1)	72 (55.4)	0.042
Smoking, *n* (%)	3 (6.5)	39 (29.2)	0.001
Acetylsalicylic acid, *n* (%)	21 (45.6)	60 (46.1)	0.546
Ace-inhibitors, *n* (%)	15 (32.6)	45 (34.6)	0.477
AT II receptor blocker	6 (13.1)	25 (19.2)	0.239
Insulin, *n* (%)	2 (4.3)	5 (3.8)	0.587
Oral anti-diabetic drugs, *n* (%)	6 (13.1)	30 (23.1)	0.106
Statin, *n* (%)	7 (15.2)	27 (20.8)	0.278
Glycemia (mg/dl)	129.65 ± 21.02	129.22 ± 27.64	0.924
Cholesterol (mg/dl)	181.2 ± 21.4	197.7 ± 21.1	0.001
LDL-cholesterol (mg/dl)	111.57 ± 21.65	123.83 ± 20.43	0.010
Triglycerides (mg/dl)	163.91 ± 23.63	181.79 ± 18.06	0.001
LDH (unit/l)	367.83 ± 37.41	375.46 ± 33.32	0.126
Creatinine (mg/dl)	0.92 ± 0.22	1.04 ± 0.14	0.001
Fibrinogen (g/l)	3.68 ± 0.37	3.75 ± 0.33	0.198
White cells count (10^9^/l)	15.76 ± 1.27	12.49 ± 4.82	0.002
Lymphocyte (10^9^/l)	1.76 ± 0.20	1.80 ± 0.14	0.130
Virus load CT, *n*	26.02 ± 2.22	/	/
Thrombus virus load CT, *n*	25.46 ± 8.68	/	/
Ejection fraction (%)	41.49 ± 1.82	47.25 ± 3.55	0.003
LAD, *n* (%)	31 (67.4)	74 (56.9)	0.091
LMS, *n* (%)	8 (17.4)	15 (11.3)	0.145
Cx, *n* (%)	5 (10.9)	13 (10.0)	0.520
RCA, *n* (%)	12 (26.1)	39 (30.0)	0.349
GP IIb/IIIa inhibitor, *n* (%)	25 (54.3)	19 (14.6)	0.006
Multi-vessel PCI, *n* (%)	10 (21.7)	11 (8.5)	0.020
Thrombus dimension, mm^2^	4.21 ± 1.12	2.71 ± 0.42	0.001
Modified thrombus G 5, *n* (%)	41 (89.1)	30 (23.1)	0.001
Multivessel thrombus, *n* (%)	16 (34.8)	12 (9.2)	0.001
Post-PPCI TIMI 3, *n* (%)	43 (93.5)	124 (95.4)	0.432
Blush grade 2–3, *n* (%)	12 (26.1)	127 (97.7)	0.001
Total heparin dose, U	12777.78 ± 356	10137.55 ± 299	0.047
Door-to-balloon time (min)	54 (38–72)	51 (35.3–57.9)	0.345
Average ACT	268.7 ± 67.2	271.6 ± 59.3	0.167
*MACE*			
Death, *n* (%)	4 (8.7)	2 (1.5)	0.041
Non-fatal AMI, *n* (%)	6 (13.0)	4 (3.1)	0.021
Heart failure, *n* (%)	4 (8.7)	3 (2.3)	0.077

Data are means ± SD or *n* (%)

BMI, body mass index; CT, cycle threshold; LAD, left anterior descending artery; LMS, left main coronary artery; MACE, major adverse cardiac events; Cx, circumflex artery; RCA, right coronary artery; ACT, activated clotting time; AMI, acute myocardial infarction

### Procedural characteristics

All patients underwent a primary PCI procedure and TA in both groups. Time from symptoms to reperfusion and ECG presentations were similar in both groups. Median door to balloon times was within 66 ± 16 min and similar for both groups. All patients received a loading dose of aspirin 300 mg and either clopidogrel 600 mg or ticagrelor 180 mg before the procedure. All patients then received 100 mg aspirin per day plus either 75 mg clopidogrel per day or 90 mg ticagrelor twice-daily maintenance therapy. Interestingly, there were no differences between the ASAP group and the SANE group in the % stenosis of the coronary lesion after thrombectomy but before stenting. There was evidence of higher thrombogenicity in the ASAP patients with significantly higher rates of modified thrombus grade 5 (Table 1) and thrombus dimension (Fig. 1a). Myocardial blush grade and left ventricular ejection fraction were significantly lower in the ASAP patients than the SANE patients (Table 1). In keeping with this, there was significantly greater use of GP IIb/IIIa inhibitors (*p* < 0.0001), and to reach the similar ACTs total dose of heparin was higher in the ASAP group (Table 1). In all patients, thrombus dimension correlated with ejection fraction (*r* = 0.475, *p* < 0.001). In asymptomatic patients, thrombus viral load correlated with thrombus dimension (*r* = 0.365, *p* < 0.001), hs-Troponin levels (*r* = 0.3414, *p* < 0.001) and ejection fraction (*r* = 0.286, *p* < 0.001).

### In-hospital outcomes

After PPCI, a blush grade post-PPCI 2–3 was present in only 26.1% of ASAP vs. 97.7% of SANE STEMI patients (*p* < 0.001). Kaplan–Meier analysis in ASAP STEMI patients during hospitalization following PPCI showed a significantly lower in-hospital survival for MACE than in SANE STEMI patients (*p* < 0.001; Figure 2 D). Moreover, among MACE outcomes, death and nonfatal AMI were higher in ASAP than in SANE patients (Table 1). In all study population (176), multivariate linear regression analysis, with thrombus dimension as the dependent variable, evidenced that SARS-CoV-2 infection and metabolic risk factors, as a composite score, were independent predictors of thrombus dimension (Table 2). The predictive role of SARS-CoV-2 infection and thrombus dimension on MBG outcome was tested in multiple logistic regression analyses (Table 3). In the model A, for all the study population (ASAP and SANE STEMI patients, *n* = 176), the thrombus dimension revealed a main independent effect MBG (OR 0.399, 95% CI [0.241–0.661], *p* = 0.001). (Table 3). In the model B, including thrombus viral load and thrombus dimension in ASAP STEMI patients (*n* = 46), thrombus dimension was a significant predictor of poor MBG independently of age, sex, smoking, hypertension, and metabolic risk factor composite score (OR 0.010, 95% CI [0.001–0.130], *p* = 0.001). (Table 3). In the model C, including thrombus viral load (in patients with SARS-CoV-2 infection) and excluding thrombus dimension in ASAP STEMI patients (*n* = 46), the thrombus viral load was significant predictor of poor MBG independently of age, sex, smoking, hypertension, and metabolic risk factor composite score (OR 0.007, 95% CI [0.000–0.014], *p* = 0.008). (Table 3).

**Table 2:** Multivariate linear regression analysis with thrombus dimension as dependent variable (*n* = 176)

Variables	*B*	Error std.	Beta	*t*	*p*
Age	.009	.008	.081		
Sex	− .271	.139	− .136	− 1.943	.054
SARS-CoV-2 infection	1.439	.170	.674	8.451	.000
Metabolic risk factor composite score	− .051	.023	− .144	− 2.234	.027
Hypertension	.064	.119	.034	.539	.591
Smoking	− .091	.142	− .041	− .642	.522

**Table 3:** Multiple logistic regression analyses with Blush grade (not vs. yes) as dependent variable for all study population (model A, *n* = 176) and for Asymptomatic SARS-COV-2 Positive patients (model B, *n* = 46).

	OR	95% CI	*P* value
*MODEL A*			
Age	1.067	0.999–1.140	0.053
Sex	0.340	0.089–1.299	0.115
Metabolic risk factor Composite score	0.195	0.030–4.320	0.198
Hypertension	0.441	0.039–0.644	0.125
Smoking	0.582	0.144–2.345	0.446
Thrombus dimension	0.399	0.241–0.661	0.001*
*MODEL B*			
Age	0.873	0.780–0.977	0.018*
Sex	0.116	0.011–1.240	0.075
Metabolic risk factor Composite score	0.243	0.023–2.544	0.238
Hypertension	0.914	0.220–3.804	0.902
Smoking	1.782	0.254–12.488	0.561
Thrombus dimension	0.010	0.001–0.130	0.001*
Thrombus viral load	1.257	0.646–2.443	0.501
*MODEL C*			
Age	0.876	0.784–0.978	0.018*
Sex	8.110	0.778–8.452	0.080
Metabolic risk factor Composite score	4.171	0.404–4.305	0.230
Hypertension	1.098	0.268–4.493	0.897
Smoking	0.602	0.083–4.370	0.615
Thrombus viral load	0.007	0.000–0.014	0.008*

OR, odds ratio; CI, confidence of interval. *P* value < 0.05 is statistical significant, marked with *.

**Fig. 3:**
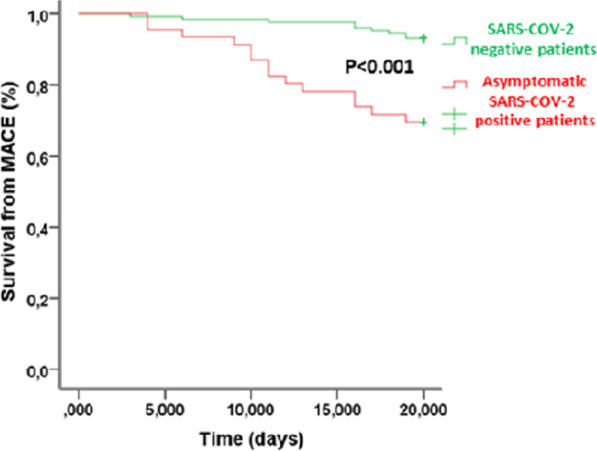
Risk-adjusted Kaplan–Meier analysis curves showing survival from MACE through follow-up in STEMI patients in asymptomatic SARS-COV-2 positive and SARS-COV-2 negative patients.

Indeed, categorizing ASAP patients (*n* = 46) by gender, female had higher thrombus viral load (15.53 ± 4.5 vs 30.25 ± 5.51 CT; *p* < 0.001) and thrombus dimension (4.62 ± 0.44 vs 4.00 ± 1.28 mm^2^; *p* < 0.001) compared to male subjects. In contrast, no difference by gender in thrombus dimensions was found in all study population (*n* = 176). Finally, in Figure 3 we showed the Kaplan curves for survival from MACE through follow-up in STEMI patients in ASAP vs. SANE patients.

In addition, 2 ASAP (4.3%) vs. 3 SANE patients (2.6%) had mechanical complications (*p* < 0.05), as ventricular free-wall rupture (1 (50%) vs. 2 (66.7%); *p* < 0.05), and ventricular septal rupture (1 (50%) vs. 1 (33.3%); *p* < 0.05).

However, in ASAP vs. SANE patients we had significant difference in hospital stay (4.8 ± 1.1 vs. 2.9 ± 0.7 days; *p* < 0.05), and need for mechanical circulatory support (*n* 3 (6.5%) vs. 3 (0.2%) patients, *p* < 0.05. Indeed, ASAP vs. SANE patients had a significant different use of intra-aortic balloon pump, IABP (1/3 (33.3%) vs. *n* 1/3 (33.3%), *p* > 0.05). No difference was found regards the use of veno-arterial-venous extracorporeal membrane oxygenation (VAV ECMO: 2/3 (66.6%) vs. *n* 2/3 (66.6%) patients, *p* > 0.05) and IABP + VA ECMO (1/3 (33.3%) vs. *n* 1/3 (33.3%) patients, *p* > 0.05). Comparing ASAP vs. SANE, we had a significant difference regards the need for intensive care (9 (19.6%) vs. 8 (6.1%) patients; *p* < 0.05), and we had not difference regards the kidney injury (8/46 (17.4%) vs. 19/130 (14.6%) patients; *p* > 0.05). In addition, 3 ASAP (6.5%) vs. 3 SANE patients (2.3%), (*p* < 0.05) had acute cardiogenic shock.

Regards further cardiac manifestation, comparing ASAP vs. SANE we had: (1) 2 (4.3 %) vs. 4 (3.1%) patients with pericarditis, (*p* > 0.05); (2) 2 (4.3%) vs. 3 (2.3%) patients with pericardial effusion, (*p* < 0.05); (3) arrhythmias: 16 (34.8%) vs. 31 (23.8%) patients with ventricular tachycardia, (*p* < 0.05); 41 (89.1%) vs. 102 (78.5%) patients with premature beats, (*p* < 0.05); 27 (58.7%) vs. 73 (56.1%) patients with paroxysmal tachycardia, (*p* > 0.05); and 20 (40.5%) vs. 39 (30%) patients with atrial fibrillation, (*p* < 0.05). The main reasons for death comparing ASAP (*n* 4) vs. SANE (*n* 2) patients were cardiogenic shock (3 (75%) vs. 1 (50%) patient; *p* < 0.05), and anoxic brain damage (1 (25%) vs. 1 (50%) patient; *p* < 0.05).

## Discussion

This study represents the first comparative data to describe the coronary thrombus viral load and dimension in ASAP patients presenting with STEMI. This analysis demonstrates increased thrombus dimension in ASAP STEMI patients compared with STEMI patients who are not infected. Interestingly, 39 (84.9%) of asymptomatic patients with SARS-COV-2 positive respiratory specimens also had coronary thrombus specimens positive for SARS-COV-2. These observations fit a higher incidence of multiple thrombotic culprit lesions as well as lower MBG. Consistent with this, lower left ventricular systolic function and increased troponin levels were observed in ASAP STEMI patients despite similar median ischemia times. ASAP STEMI patients more often had blood abnormalities reflecting a systemic inflammatory response (elevated D-dimers and C-reactive protein levels) compared with SANE patients. Although SANE STEMI patients more often had hypertension, and obesity as well as were older, higher rates of poor blush grade (< 2) were seen in ASAP patients, suggesting the extent of damage of the microvascular bed [12]. In fact, despite more than 90% of ASAP STEMI patients presented restoration of epicardial blood flow after PPCI (TIMI grade flow 3), only 26% of ASAP patients had blush grade post-PPCI 2–3. In addition, the female gender caused in ASAP patients a higher thrombus viral load and higher thrombus dimension as compared to male subjects. Notably, the MBG is associated with ST-segment elevation resolution, enzymatic infarct size, left ventricular function, and long-term mortality [17]. Interestingly, there were no differences between the ASAP group and the SANE group in the % stenosis of the coronary lesion after thrombectomy but before stenting. This observation suggests that in agreement with the difference in thrombus viral load, the lesions appear different about thrombus burden, but not in the degree of vessel stenosis, suggesting that ASAP and SANE lesions were only different regarding coagulation burden. Accordingly, we observed a reduced survival from in-hospital mortality, nonfatal AMI, and heart failure were seen in ASAP patients compared to SANE patients. Although these data were obtained in a relatively small number of ASAP STEMI patients, the reduced survival may be supported as an outcome by the blush grade evaluation that evidenced reduced perfusion in the microvascular bed of ASAP patients. Intriguingly, coronary thrombus viral load was found to correlate with thrombus dimension and troponin T levels, suggesting an important role of SARS-COV-2 thrombus colonization in poorer outcomes of ASAP STEMI patients. Here we describe coronary thrombus assessment features such as thrombus viral load and dimension, which raise the suspicion of the active role of SARS-COV-2 in the pro-thrombotic state of ASAP STEMI patients. Since STEMI was the first manifestation of the disease in this cohort and ASAP patients had lower cardiovascular risk factors than SANE patients, our data suggest that presentation of STEMI might itself be considered a thrombotic complication of SARS-COV-2 dissemination through endothelial cells and thrombus. Accordingly, thrombus viral load was a significant predictor of thrombus dimension independently of age, sex, smoking, hypertension, and the compound score of metabolic risk factors. However, despite the evidence of classical cardiovascular risk factors, and/or of the female gender as main determinant of higher thrombus viral load and thrombus dimension, here we might suggest that the thrombus viral load is itself the main determinant of thrombus dimension and lowest MBG in ASAP patients. Thus, this might explain a mechanistic relation between SARS-CoV-2 infection and the intra-coronary thrombus burden in STEMI patients. Indeed, the predictive role of SARS-CoV-2 infection and thrombus dimension on blush grade outcome was tested in multiple logistic regression analyses suggesting that SARS-CoV-2 infection and thrombus dimension were significant predictors of adverse clinical outcome in STEMI patients. Taken together, these data raise the doubt that asymptomatic status in SARS-COV-2 positive patients might affect STEMI outcomes as it may have major implications in patient management. As background for these associations, pre-COVID data regarding the influenza virus suggest that patients with acute respiratory infections were at significantly elevated risk for developing atherosclerotic plaque rupture leading to myocardial infarction, the profound inflammatory response, and hemodynamic changes [18]. These previously reported features are associated with higher coronary thrombus burden and poorer outcomes in COVID-19 cohorts with STEMI [19–21]. COVID-19 infection is associated with a pro-thrombotic state [22]. The occurrence of venous thrombus-embolic complications, both clinically apparent and subclinical, appears to be an important manifestation of the COVID-19 and related to disease severity and outcome [23].

Indeed, SARS-CoV-2 generates a prothrombotic state with higher thrombotic complications such as deep venous thrombosis [24]. This effect is mainly mediated via increased Neutrophil extracellular traps that have potential to propagate inflammation and microvascular thrombosis in patients with SARS-CoV-2 infection [25]. Furthermore, SARS-CoV-2 is able to potentiate a hyper-coagulable state via activation of the contact and tissue factor pathways [26]. Then, the SARS-CoV-2–platelet interactions result in platelet activation and degranulation in COVID-19, further potentiating the pro-thrombotic vascular milieu [27]. Moreover, the SARS-CoV-2 infection is associated with platelet hyperreactivity [27], e pro-thrombosis which may contribute to COVID-19 pathophysiology [27, 28].

In this context, authors just reported an increased thrombogenicity in acute ischemic stroke during SARS-CoV-2 infection [29]. Notably, other respiratory infections (such as community-acquired pneumonia) also increase thrombogenic potential, both regarding coagulation and platelet aggregation [30]. Thus it is not unexpected that Covid-19 increases thrombotic potential [31].

On the other hand, emerging data from large COVID-19 cohorts without STEMI [32] suggests that anticoagulation confers mortality benefit in this patient group. However, there have been no reports of increased coronary artery thrombus dimension, thrombus SARS-COV-2 colonization, and poorer blush grade outcomes in ASAP patients presenting with STEMI. Mechanisms that trigger a presentation with STEMI and its associated higher arterial thrombus burden in ASAP patients are unknown. Relative to venous thromboembolism, arterial thrombus formation is more likely to be due to platelet activation and endothelial dysfunction. SARS-CoV-2 causes a systemic inflammatory response, leading to endothelial and hemostatic activation, including the activation of platelets and the coagulation cascade [18]. In keeping with this, the data presented here show significantly higher rates of CPR, D-dimer, and Troponin I levels in the ASAP patients suggesting that this condition may also confer an increased risk of poorer blush grade outcome in ASAP patients. Mechanisms for this might include increased endothelial dysfunction or their effects on the immune system [30–35]. Whether these alterations may be responsible for poor outcomes in ASAP patients is a point to be confirmed with further studies. However, the similar viral load evidenced in asymptomatic and COVID-19 patients could raise the doubt that, also in asymptomatic patients, SARS-COV-2 can produce endothelial damage and increase coagulation.

### Study limitations

It is a relatively small observational study and has all the limitations of this type of analysis, including bias and confounding potential. Moreover, the short duration of follow-up did not fully explain the effects of SARV-COV-2 in STEMI outcomes in ASAP patients. On the other hand, our objective was to evaluate thrombus viral load and thrombus burden as well their impact on microvascular bed perfusion in the infarct area (Blush grade, MBG) in asymptomatic SAR-COV-2 positive compared to SARS-COV-2 negative STEMI patients, and not to evaluate the effects of thrombus aspiration and/or treatment and/or a drug on clinical outcomes, objectives that require a randomized trial. Moreover, patient selection is in accordance with the routine clinical practice, as patients underwent thrombus aspiration based on 2017 AHA guidelines [36] and ESC 2017 [37] to manage the patient with STEMI. Accordingly, the percentage of patients undergoing thrombus aspiration in our study (176 of 474, 37%) agrees with previous and more numerous observational studies [36–39]. Another limitation of the study was the not fully evaluation of inflammatory markers as pro-inflammatory cytokines.

## Conclusion

In our article the ASAP vs. SANE were younger with lower BMI, lower rate of hypertension and smoking (*p* < 0.05). Thus, ASAP vs. SANE evidenced less typical cardiovascular risk factors during STEMI. To date, from one side the ASAP could have a lower metabolic risk factor composite score, that could impact on the triage strategies for patients presenting with STEMI in the post-COVID era. On the other hand, this could reinforce the study hypothesis that the SARS-CoV-2 isolation from the nasopharyngeal swab in ASAP patients with STEMI could itself characterize a cohort of patients that could evidence the SARS-CoV-2 inside the intra coronary thrombus, and that could have higher thrombus dimension independently of risk factors (*p* < 0.005). Furthermore, the ASAP patients were also associated with poorer MBG and worse outcomes as compared to SANE patients presenting with STEMI. Notably, despite the greater use of GP IIb/IIIa inhibitors and heparin in the ASAP vs. SANE STEMI patients, at clinical discharge (after PCI) we used the

same anti-platelets and anti-thrombotic therapies for both cohorts. Indeed, by the actual lack of specific anti-thrombotic therapies recommendation for post-STEMI patients with SARS-CoV2, we did not modify the post-discharge medications protocols in SARS-CoV-2 positive patients with STEMI and treated by PCI. Thus, we do not suggest a different post-STEMI/PCI management approach for ASAP vs. SANE STEMI patients to prevent thrombotic complications.

Finally, taken together our data could indicate that SARS-CoV-2 determines a different pathophysiological mechanism leading to STEMI compared to other patients. In this context, we would remark the concept to observe a new kind of disease which should treated in a slightly different way compared to normal STEMI management.

Indeed, the strong signal towards significantly higher thrombus viral load and higher thrombus dimension is a novel finding that raises the question of more aggressive anti-thrombotic therapy in patients at high risk for cardiovascular diseases, as diabetic, hypertensive, and dyslipidemic ASAP patients. These observations may be helpful for considering the asymptomatic SARS-COV-2 positive condition as a clinical state that may influence atherosclerotic patients' cardiovascular outcomes. Further studies are needed to confirm this critical finding.

## Data Availability

The datasets used and analyzed during the current study are available from the corresponding author on reasonable request.
